# Crystal Structures of New Ammonium 5-Aminotetrazolates

**DOI:** 10.3390/cryst4040439

**Published:** 2014-11-19

**Authors:** Martin Lampl, Robert Salchner, Gerhard Laus, Doris E. Braun, Volker Kahlenberg, Klaus Wurst, Gerda Fuhrmann, Herwig Schottenberger, Hubert Huppertz

**Affiliations:** 1 Institute of General, Inorganic and Theoretical Chemistry, University of Innsbruck, 6020 Innsbruck, Austria; 2 Institute of Pharmacy, University of Innsbruck, 6020 Innsbruck, Austria; 3 Institute of Mineralogy and Petrography, University of Innsbruck, 6020 Innsbruck, Austria

**Keywords:** 5-aminotetrazole, cystamine, tetramethylammonium, tetramethylguanidine

## Abstract

The crystal structures of three salts of anionic 5-aminotetrazole are described. The tetramethylammonium salt (*P*$\overset{‒}{1}$) forms hydrogen-bonded ribbons of anions which accept weak C–H⋯N contacts from the cations. The cystamine salt (*C*2/*c*) shows wave-shaped ribbons of anions linked by hydrogen bonds to screw-shaped dications. The tetramethylguanidine salt (*P*2_1_/*c*) exhibits layers of anions hydrogen-bonded to the cations.

## 1. Introduction

Due to its amphiprotic nature [1,2], 5-aminotetrazole has served as a cation [3,4] or as an anion in energetic salts [5]. Crystal structures of several alkali metal 5-aminotetrazolates have been reported [6]. Organic salts containing 5-aminotetrazole as anion have been first described more than six decades ago [7]. Continuing our interest in nitrogen-rich heterocycles [8-11], we describe here the preparation and crystal structures of three new 5-aminotetrazolates of organic cations. These salts are of interest as blowing agents [12] and as alternative precursors for nitridosilicates [13-15]. Following the structural characterization, the new compounds were examined by DSC and TGA in order to assess their suitability for those purposes.

## 2. Results and Discussion

**Figure 1:**
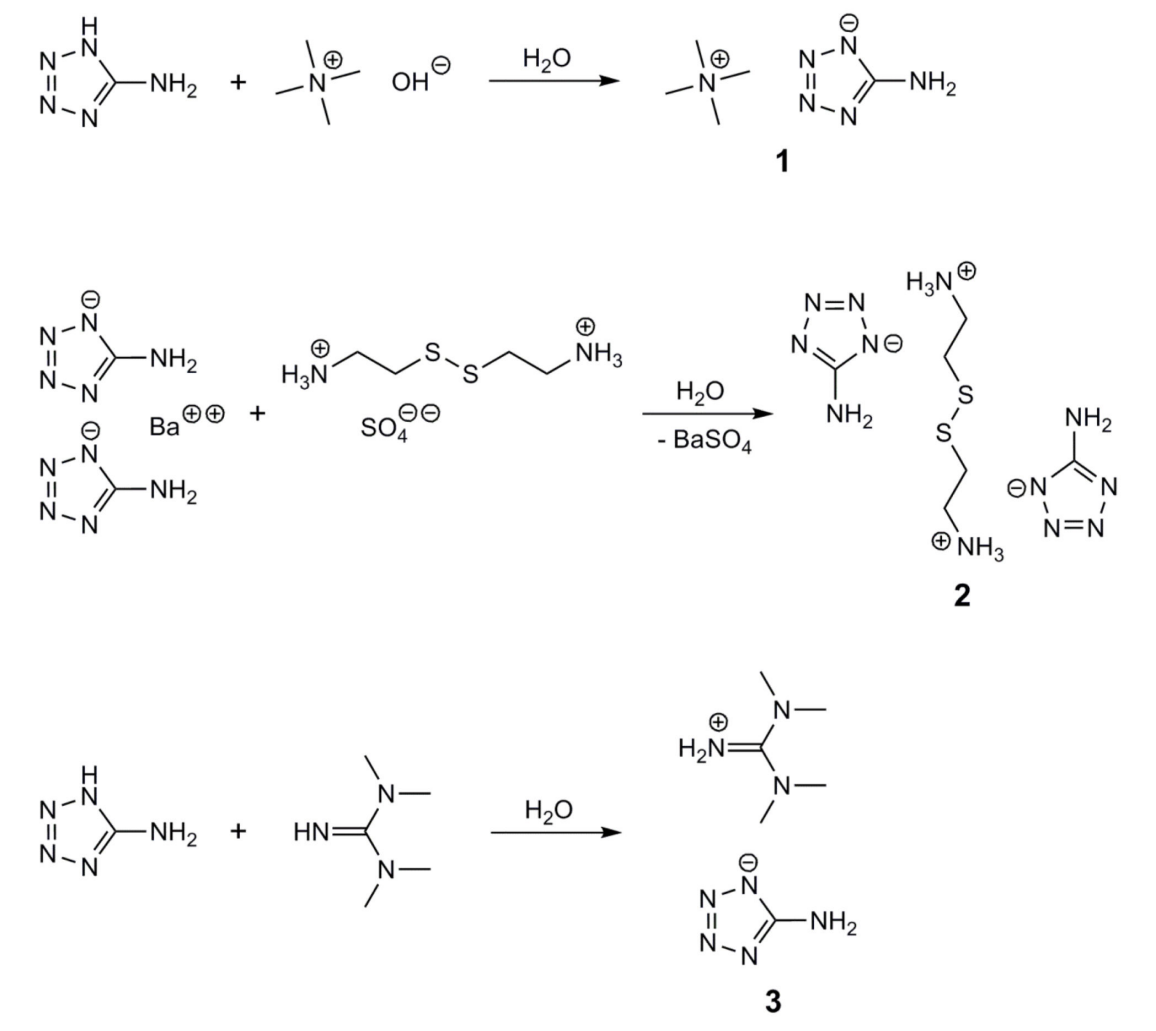
Synthesis of the 5-aminotetrazolates **1**–**3**.

Three synthetic methods were employed to generate the required 5-aminotetrazolate anion (Figure 1), *i.e.*, (a) deprotonation of 5-aminotetrazole by an organic hydroxide; (b) ion metathesis of an organic sulfate with barium 5-aminotetrazolate; and (c) direct reaction of 5-aminotetrazole with a strong organic base.

The crystal data and details of the structure refinements for tetramethylammonium 5-aminotetrazolate (**1**); cystamine bis(5-aminotetrazolate) (**2**); and 1,1,3,3-tetramethylguanidinium 5-aminotetrazolate (**3**) are summarized in Table 1.

### 2.1. Tetramethylammonium 5-Aminotetrazolate (**1**)

**Figure 2:**
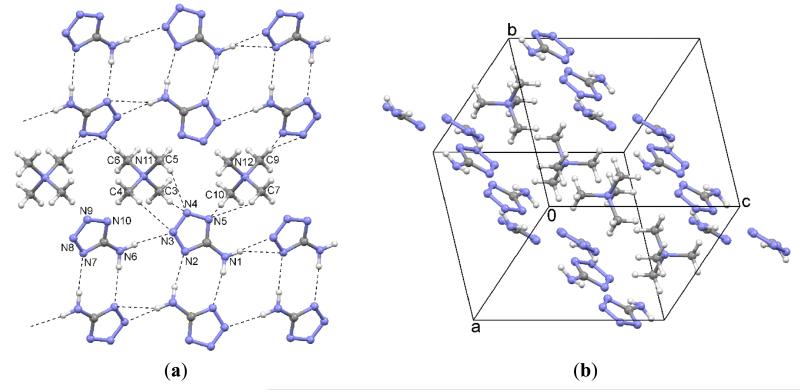
(**a**) Interactions in the crystal structure of **1**; (**b**) Packing diagram of **1**.

**Figure 3:**
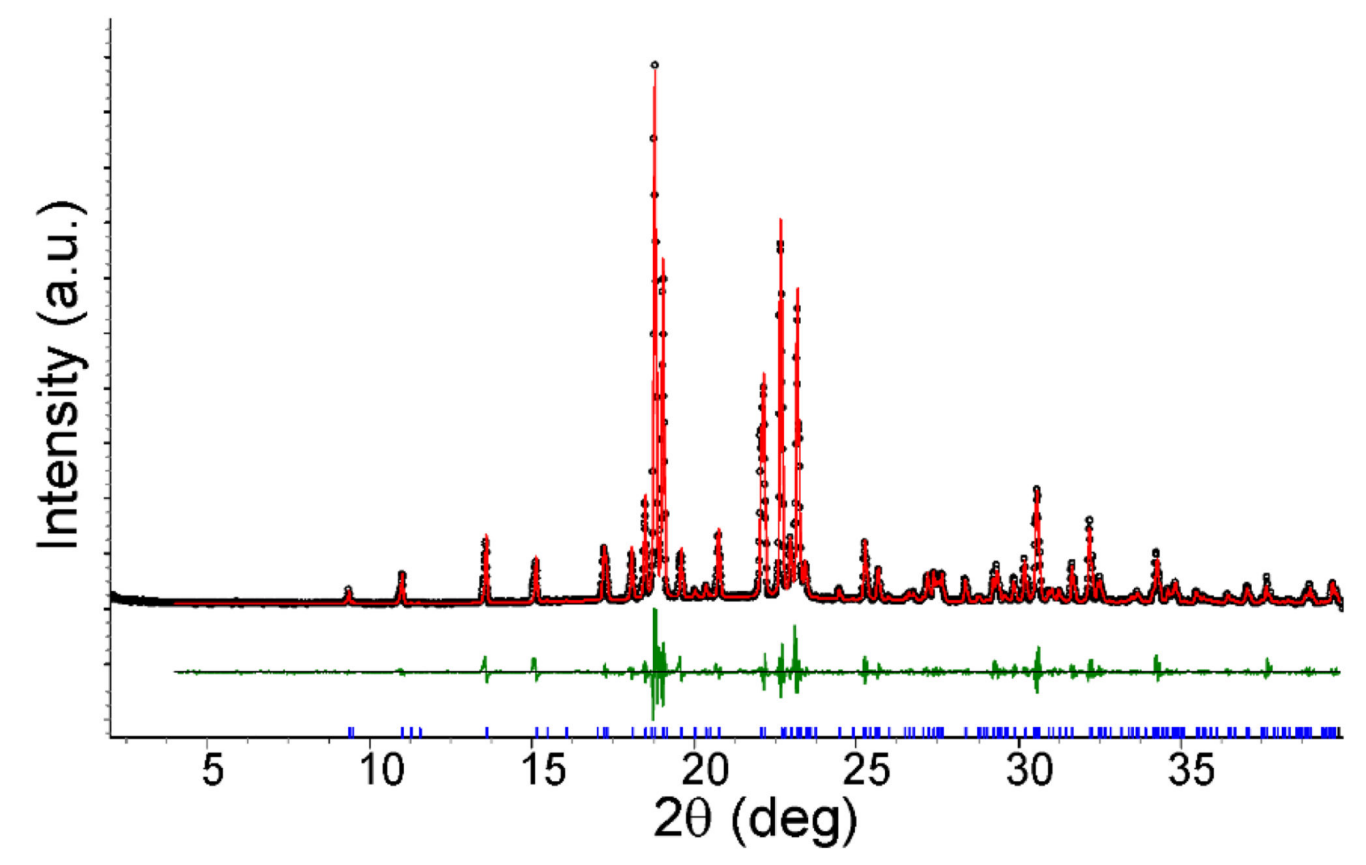
Pawley fit (*R*_wp_ = 12.11%, *R*_exp_ = 5.47%, *R*_p_ = 8.66%, *gof* = 2.21) of the powder X-ray diffraction (PXRD) data of **1** with a model calculated from the structure parameters derived from the single crystal structure. Black dots indicate raw data, while the red line indicates the calculated model. Tick marks (blue) are the 2θ positions for the *hkl* reflections. The difference curve is shown in green.

Two independent ion pairs are observed in the asymmetric unit. The tetrazolate anions arranged in ribbons parallel to the ($\overset{‒}{1}$ 11) plane accept weak C–H⋯N contacts from the quaternary cations which are aligned in rows parallel to the [1 $\overset{‒}{1}$ 1] direction (Figure 2a). The rows of cations are located sideways between the ribbons and above/below the plane of the ribbons, as displayed in Figure 2b. The hydrogen bond parameters are collected in Table 2. Phase purity of the bulk sample was confirmed by Pawley fit between the experimental and the calculated powder patterns (Figure 3 and Table 3).

### 2.2. Cystamine Bis(5-aminotetrazolate) (**2**)

**Figure 4:**
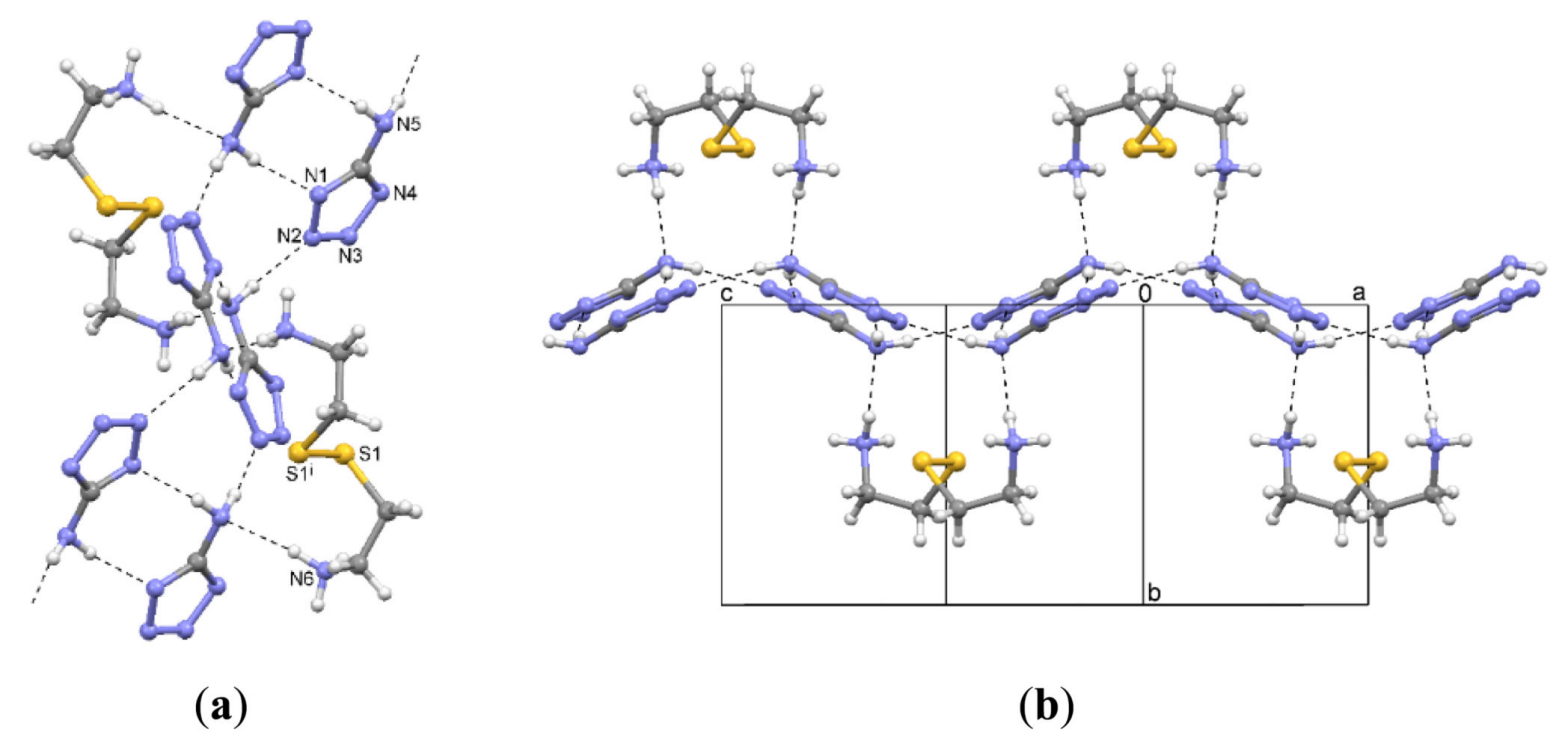
(**a**) Interactions in the crystal structure of **2**; i: −*x, y*, 3/2 – *z*; (**b**) Packing diagram.

**Figure 5:**
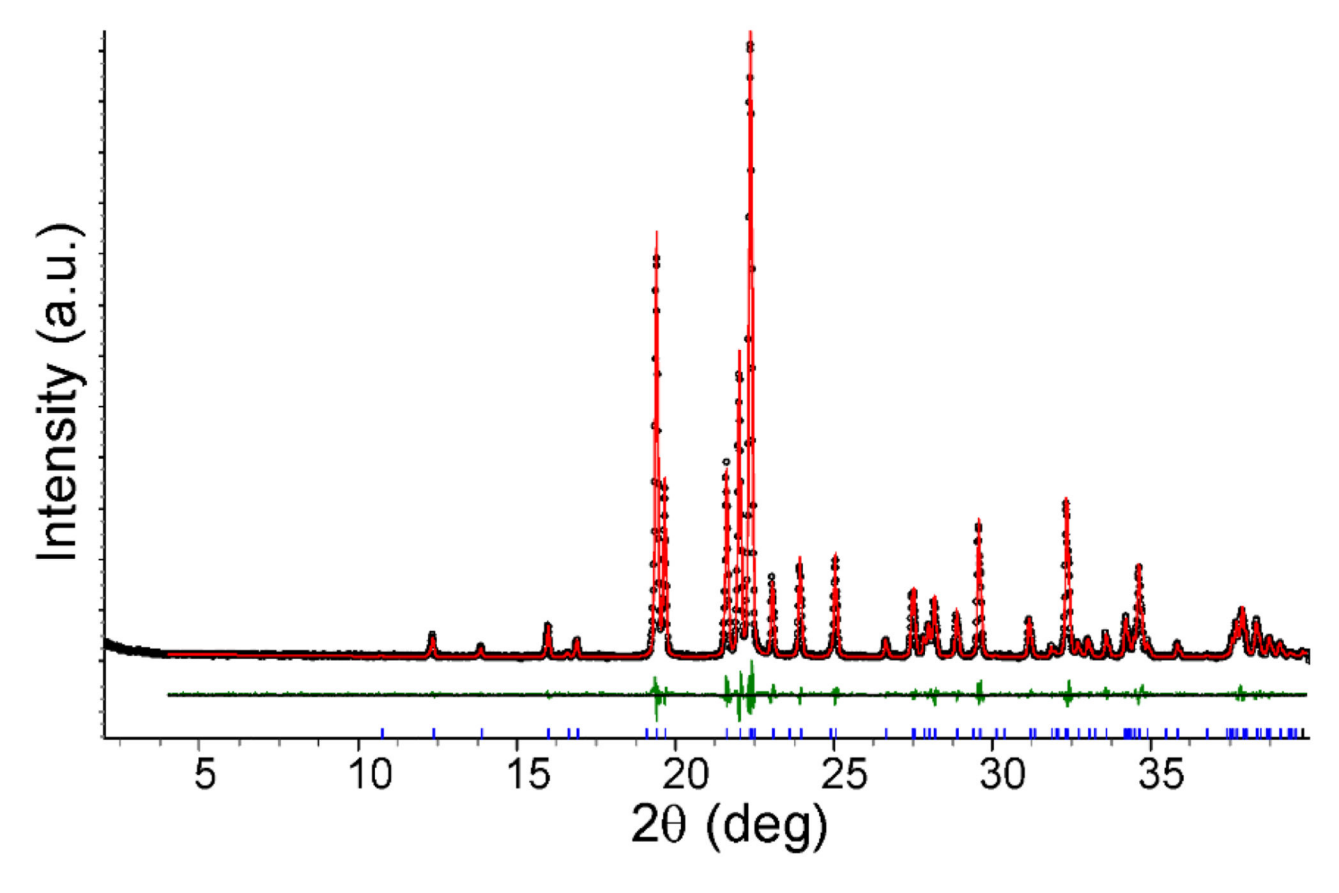
Pawley fit (*R*_wp_ = 9.04%, *R*_exp_ = 7.96%, *R*_p_ = 6.89%, *gof* = 1.14) of the PXRD data of **2** with a model calculated from the structural data of the single crystal structure determination.

The fragments of the cystamine cations are completed by a two-fold rotation axis perpendicular to the S1–S1 bond. The resulting dications are screw-shaped with a C–S–S–C dihedral angle of 89.6(1)° (Figure 4a). The anions form hydrogen-bonded wave-shaped ribbons parallel to the *ac* plane to which the cations are linked by hydrogen bonds (Figure 4b). The Pawley fit is depicted in Figure 5, and the corresponding results are shown in Table 4.

### 2.3. 1,1,3,3-Tetramethylguanidinium 5-Aminotetrazolate (**3**)

**Figure 6:**
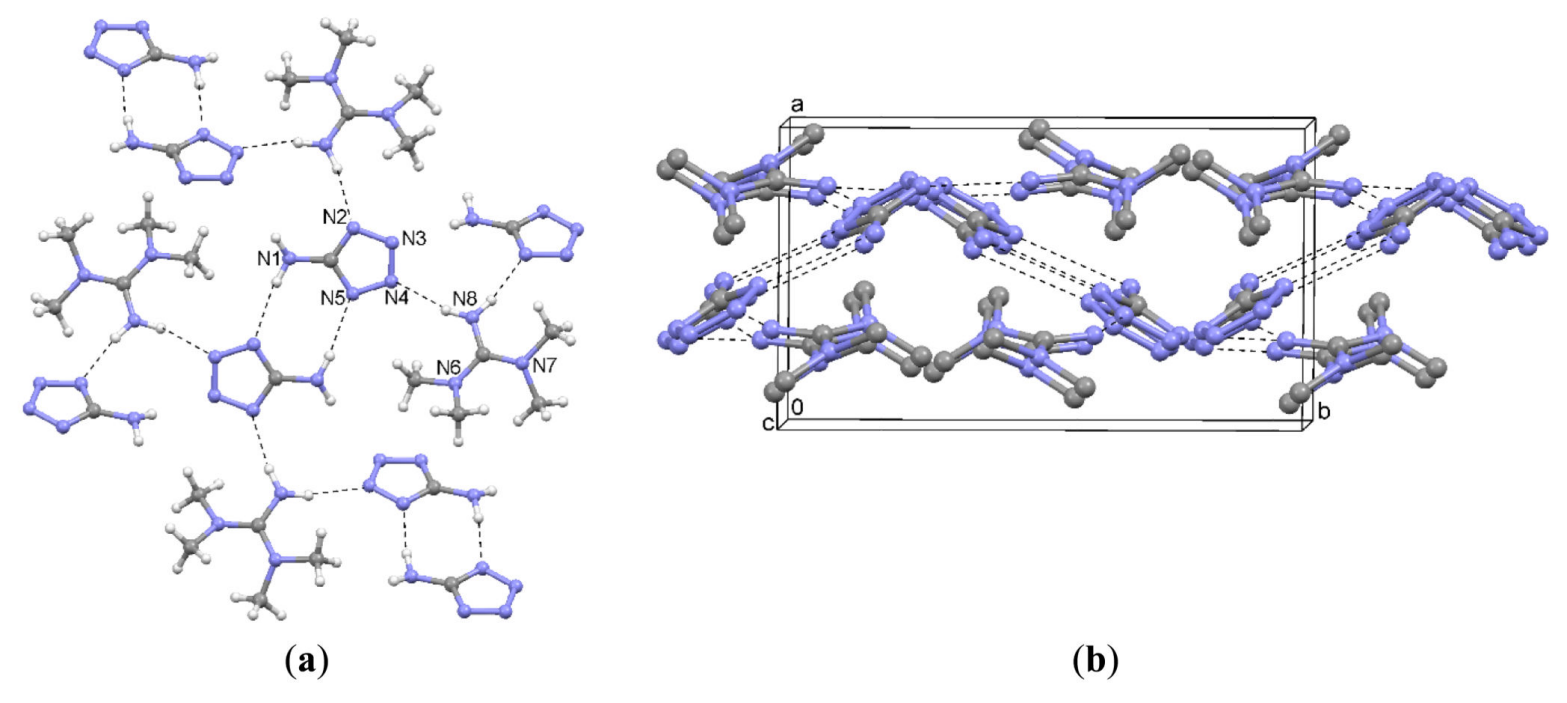
(**a**) Interactions in the crystal structure of **3**; (**b**) Packing diagram of **3**; hydrogen atoms are omitted for clarity.

**Figure 7:**
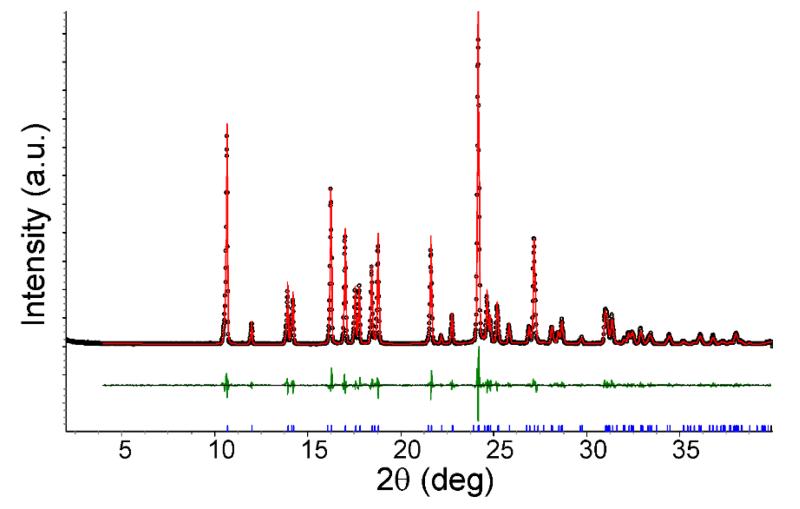
Pawley fit (*R*_wp_ = 10.03%, *R*_exp_ = 5.57%, *R*_p_ = 7.80%, *gof* = 1.80) of the PXRD data of **3** with a model calculated from the structural data of the single crystal structure determination.

The crystal structure of **3** is composed of hydrogen-bonded layers of anions and cations (Figure 6a) arranged parallel to the *bc* plane (Figure 6b). The Pawley fit is shown in Figure 7, and the corresponding results are collected in Table 5.

### 2.4. Differential Scanning Calorimetry (DSC) and Thermogravimetric Analysis (TGA)

**Figure 8:**
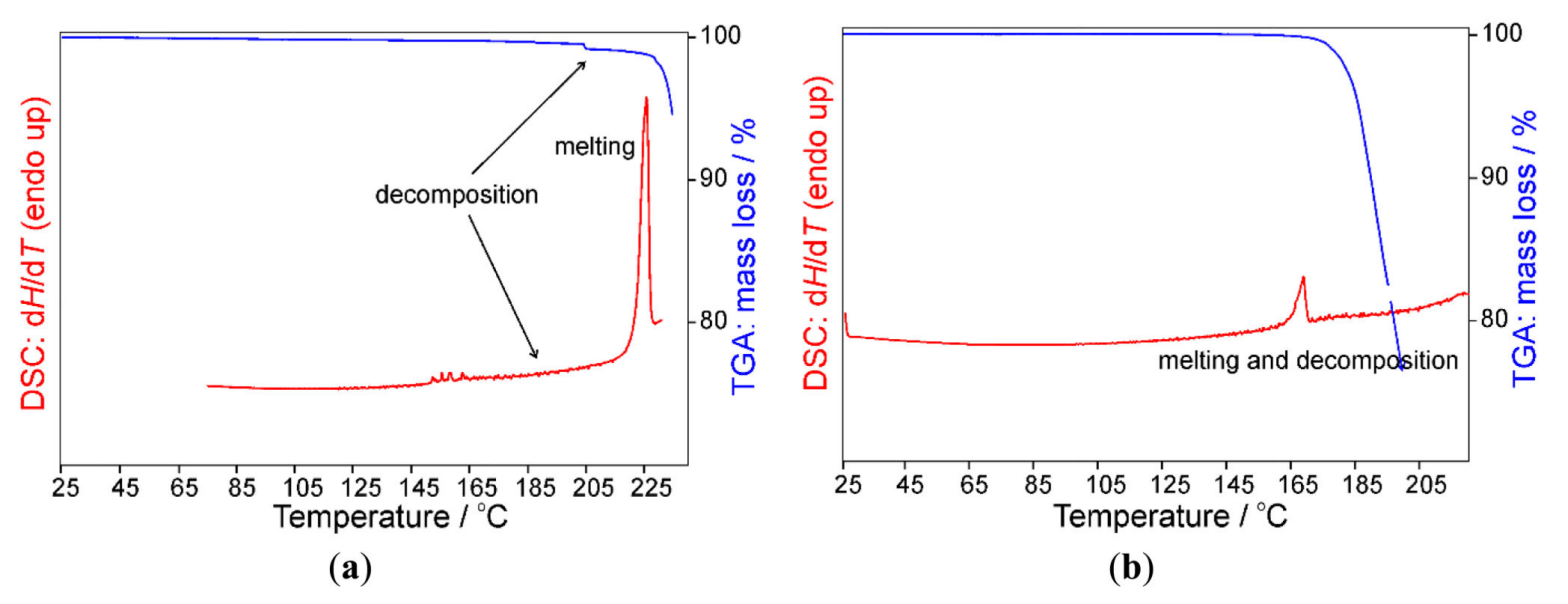

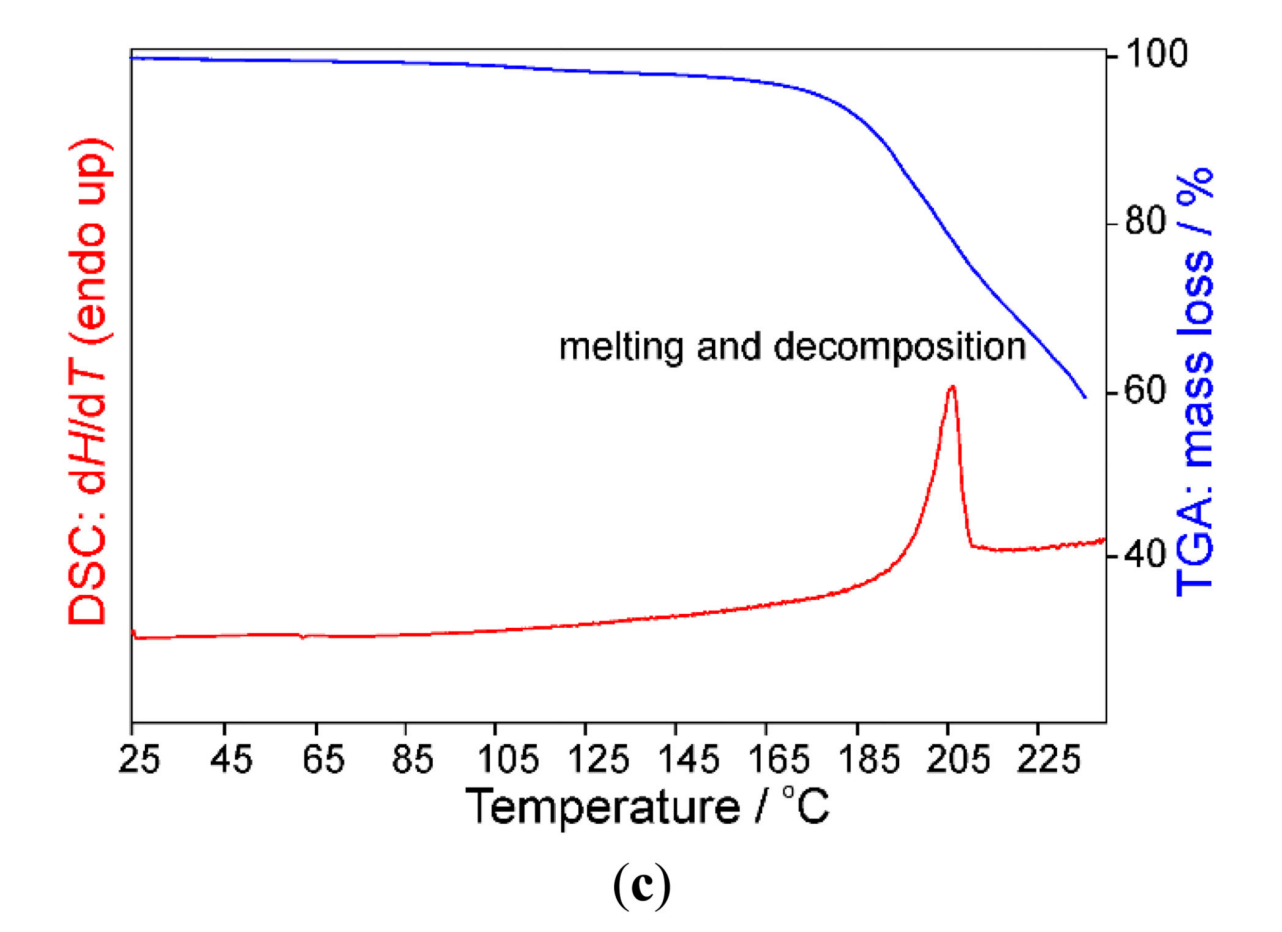
Differential Scanning Calorimetry/Thermogravimetric Analysis (DSC/TGA) thermograms of (**a**) **1**; (**b**) **2**; and (**c**) **3**.

Thermoanalysis of the 5-aminotetrazolates **1**–**3** showed an extensive decomposition with loss of mass around the melting temperature. The pertinent thermograms are depicted in Figure 8. Since the decomposition is not exothermic, the new compounds are not “energetic salts” in a strict sense, but appear to be suitable as temperate blowing agents.

## 3. Experimental Section

Barium 5-aminotetrazolate tetrahydrate was prepared according to a published procedure [16]. All other chemicals were purchased from Sigma-Aldrich, St. Louis, MO, USA (European affiliate, Steinheim, Germany). NMR spectra were recorded with a Bruker Avance DPX 300 spectrometer (Billerica, MA, USA). IR spectra were obtained with an Alpha FT (Bruker) instrument. Elemental analyses were conducted at the University of Vienna, Austria. DSC was performed with a DSC 7 (Perkin-Elmer, Norwalk, CT, USA) applying a heating rate of 10 °C·min^−1^. TGA was carried out with a TGA 7 system (Perkin-Elmer) at a heating rate of 10 °C·min^−1^. XRPD patterns were obtained using a X’Pert PRO diffractometer (PANalytical, Almelo, The Netherlands) equipped with a theta/theta coupled goniometer in transmission geometry, programmable XYZ stage with well plate holder, Cu*K*α_1,2_ radiation source with a focussing mirror, a 0.5° divergence slit and a 0.02° Soller slit collimator on the incident beam side, a 2 mm antiscattering slit and a 0.02° Soller slit collimator on the diffracted beam side and a 255 channel solid state PIXcel detector. The patterns were recorded, unless stated otherwise, at a tube voltage of 40 kV, tube current of 40 mA, applying a step size of 2θ = 0.013° with 400 s per step in the 2θ range between 2° and 40°. Pawley fits were performed with Topas Academic V5 (Coelho Software, Brisbane, Australia). The background was modelled with Chebyshev polynomials and the modified Thompson-Cox-Hastings pseudo-Voigt (TCHZ) function was used for peak shape fitting. Single crystal diffraction intensity data were recorded by ω scans with an Oxford Diffraction Gemini-R Ultra (Oxford Diffraction Ltd., Abingdon, Oxfordshire, UK) diffractometer (for **1** and **3**) or by ϕ and ω scans with a Nonius KappaCCD (Bruker, Billerica, MA, USA) diffractometer (for **2**) using Mo*K*α radiation. CCDC reference numbers: 1024084–1024086. These data can be obtained free of charge from The Cambridge Crystallographic Data Centre (Cambridge, UK).

### 3.1. Tetramethylammonium 5-Aminotetrazolate (**1**)

5-Aminotetrazole monohydrate (866 mg, 8.40 mmol) was added to a solution of tetramethyl-ammonium hydroxide pentahydrate (1.52 g, 8.40 mmol) in H_2_O (10 mL). The mixture was stirred at 50 °C for 20 min to give a clear solution. The solvent was removed under reduced pressure, and the residue was recrystallized from hot MeOH. Yield: 1.11 g (84%). M.p. 223–225 °C (decomposition). ^1^H·NMR (DMSO-d_6_, 300 MHz): δ 3.10 (s, 12H, CH_3_), 3.9 (br s, 2H, NH_2_) ppm. ^13^C·NMR (DMSO-d_6_, 75 MHz): δ 54.3 (4C, CH_3_), 164.1 (tetrazole) ppm. IR (neat): ṽ 3346 m, 3144 w, 3030 w, 1508 s, 1486 s (ν C = N), 1447 m, 1198 w, 1118 m, 949 s, 892 m, 750 m·cm^−1^. C_5_H_14_N_6_ (158.20): calculated C 37.96, H 8.92, N 53.12; found C 37.67, H 9.17, N 52.97.

### 3.2. Cystamine Bis(5-Aminotetrazolate) (**2**)

Cystamine sulfate monohydrate (320 mg, 1.19 mmol) was added to a solution of barium 5-aminotetrazolate tetrahydrate (450 mg, 1.19 mmol) in H_2_O (10 mL). The mixture was stirred for 15 min, and the precipitate was removed by filtration. The filtrate was taken to dryness under reduced pressure, and the residue was recrystallized from hot H_2_O/EtOH (1:4) to yield 320 mg (83%) of the colorless product. These crystals were suitable for structure determination. M.p. 165–167 °C (decom-position). ^1^H·NMR (DMSO-d_6_, 300 MHz): δ 2.77–2.81 and 2.88–2.93 (AA‘BB’, 8H, CH_2_CH_2_), 6.3 (br, 10H, NH_2_ and NH_3_) ppm. ^13^C NMR (DMSO-d_6_, 75 MHz): δ 39.4 (CH_2_), 39.9 (CH_2_), 159.0 (tetrazole) ppm. IR (neat): ṽ 3226 w, 3127 w, 2911 w, 2852 w, 2635 w, 2542 w, 2493 w, 1538 m, 1511 s (ν C=N), 1450 m, 1404 m, 1215 w, 1136 m, 1118 m, 900 s, 813 m, 442 s·cm^−1^. C_6_H_18_N_12_S_2_ (322.42): calculated C 22.35, H 5.63, N 52.13, S 19.89; found C 22.52, H 5.63, N 52.23, S 19.82.

### 3.3. 1,1,3,3-Tetramethylguanidinium 5-Aminotetrazolate (**3**)

1,1,3,3-Tetramethylguanidine (1.4 mL, 11.2 mmol) was added to a stirred suspension of 5-aminotetrazole monohydrate (1.0 g, 9.7 mmol) in H_2_O (10 mL) to give a clear solution. The solvent was removed under reduced pressure, and the residue was vacuum-dried for 3 h to yield 1.90 g (98%) of the colorless product. Single crystals were obtained by slow evaporation of a solution in MeOH. M.p. 200 °C (decomposition). ^1^H NMR (DMSO-d_6_, 300 MHz): δ 2.88 (s, 12H, CH_3_) ppm. ^13^C·NMR (DMSO-d_6_, 75 MHz): δ 40.0 (4C, CH_3_), 161.4 (C=NH_2_), 163.8 (tetrazole) ppm. IR (neat): ṽ 3409 w, 3180 w, 2904 m, 1600 s, 1573 s, 1518 s (ν C=N), 1438 m, 1414 s, 1096 m, 1066 m, 1036 m, 998 w, 768 w, 708 m, 436 m·cm^−1^. C_6_H_16_N_8_ (200.24): calculated C 35.99, H 8.05, N 55.96; found C 36.19, H 8.26, N 55.24.

## 4. Conclusions

Three new ammonium 5-aminotetrazolates were prepared, and their crystal structures were determined. They appear to be promising candidates as gas-releasing agents, as indicated by thermal analysis.

## Figures and Tables

**Table 1:** Crystal data and structure refinement details for compounds **1**–**3**.

Compound	1	2	3
CCDC No.	1024084	1024085	1024086
Chemical formula	C_4_H_12_N·CH_2_N_5_	C_4_H_14_N_2_S_2_·2(CH_2_N_5_)	C_5_H_14_N_3_·CH_2_N_5_
*M*_r_/g·mol^−1^	158.22	322.44	200.27
Crystal size/mm^3^	0.24 × 0.16 × 0.10	0.30 × 0.30 × 0.20	0.40 × 0.40 × 0.40
Crystal system	Triclinic	Monoclinic	Monoclinic
Space group	*P* $\overset{‒}{1}$	*C*2/*c*	*P*2_1_/*c*
*a*/Å	9.1270(9)	17.4437(3)	7.5109(4)
*b*/Å	10.129(1)	7.9305(3)	12.4241(4)
*c*/Å	10.432(2)	11.1625(4)	11.6513(4)
α/°	98.86(1)	–	–
β/°	110.65(2)	110.13(1)	106.82(1)
γ/°	106.77(1)	–	–
*V*/Å^3^	828.2(2)	1449.85(8)	1040.72(7)
*Z*	4	4	4
*D*_*x*_/g·cm^−3^	1.27	1.48	1.28
η	0.09	0.38	0.09
*F*(000)/e	344	680	432
Temperature/K	173(2)	233(2)	173(2)
θ_max_/°	25.4	26.0	28.6
*h*, *k*, *l* range	−10 ≤ *h* ≤ 9 −12 ≤ *k* ≤ 12 −12 ≤ *l* ≤ 11	−21 ≤ *h* ≤ 21 −9 ≤ *k* ≤ 9 −13 ≤ *l* ≤ 13	−9 ≤ *h* ≤ 8 −14 ≤ *k* ≤ 14 −14 ≤ *l* ≤ 12
Absorption correction	multi-scan	–	multi-scan
Measured reflections	5042	4661	6125
Independent reflections (*R*_int_)	3010 (0.030)	1418 (0.026)	1897 (0.021)
Observed reflections (*I* ≥ 2σ(*I*))	2317	1285	1718
Restraints/parameters	0/224	5/112	0/148
*R*_1_, *wR*_2_ (*I* ≥ 2σ(*I*))	0.042, 0.091	0.031, 0.081	0.033, 0.083
*R*_1_, *wR*_2_ (all data)	0.062, 0.106	0.034, 0.083	0.038, 0.087
Goodness of fit	1.02	1.12	1.06
Δρ_max_, Δρ_min_/e Å^−3^	0.21, −0.19	0.21, −0.38	0.36, −0.16

**Table 2:** Hydrogen bond parameters (Å, °).

Compound	Interaction	H⋯A	D⋯A	D–H⋯A	Symmetry Operation A
**1**	N1–H12⋯N2	2.17(3)	3.043(3)	170(2)	2 − *x*, 1 − *y*, 1 − *z*
	N6–H61⋯N3	2.25(2)	3.143(2)	173(2)	*x*, *y*, *z*
	N6–H62⋯N7	2.25(2)	3.150(2)	177(2)	1 − *x*, 1 − *y*, −*z*
	N1–H11⋯N7	2.51(2)	3.385(2)	159(2)	1 + *x*, *y*, 1 + *z*
	N1–H11⋯N8	2.61(2)	3.462(3)	155(2)	1 + *x*, *y*, 1 + *z*
	C9–H⋯N9	2.562	3.511(3)	162.9	1 − *x*, −*y*, 1 − *z*
	C4–H⋯N3	2.591	3.520(2)	158.3	*x*, *y*, *z*
	C5–H⋯N4	2.592	3.474(2)	149.7	*x*, *y*, *z*
	C3–H⋯N4	2.601	3.480(3)	149.3	*x*, *y*, *z*
	C10–H⋯N5	2.616	3.497(3)	149.6	*x*, *y*, *z*
	C9–H⋯N10	2.644	3.542(3)	152.5	1 − *x*, −*y*, 1 − *z*
	C7–H⋯N5	2.652	3.524(3)	148.3	*x*, *y*, *z*
	C6–H⋯N9	2.652	3.551(2)	152.7	−*x*, −*y*, −*z*

**2**	N6–H⋯N4	1.98(2)	2.870(2)	175(2)	*x*, −*y*, −1/2 + *z*
	N6–H⋯N3	2.05(1)	2.893(2)	162(2)	*x*, *y*, *z*
	N6–H⋯N5	2.07(1)	2.958(2)	164(1)	1/2 − *x*, −1/2 + *y*, 5/2 − *z*
	N5–H⋯N2	2.16(2)	3.037(2)	174(2)	*x*, −y, 1/2 + *z*
	N5–H⋯N1	2.20(2)	3.070(2)	173(2)	1 − *x*, −*y*, 3 − *z*

**3**	N8–H⋯N2	2.04(2)	2.921(2)	169(1)	1 − *x*, −*y*, 1 − *z*
	N8–H⋯N4	2.05(2)	2.937(1)	166(1)	1 − *x*, −1/2 + *y*, 3/2 − *z*
	N1–H⋯N5	2.21(2)	3.080(2)	169(1)	1 – *x*, 1 − *y*, 1 − *z*

**Table 3:** Comparison of lattice parameters determined at 173 K (single crystal X-ray diffraction data, SCXRD) and 298 K (powder X-ray diffraction data).

1	SG	*D*_*x*_/g·cm^−3^	*a*/Å	*b*/Å	*c*/Å	α/°	β/°	γ/°
SCXRD	*P* $\overset{‒}{1}$	1.27	9.1270(9)	10.129(2)	10.432(2)	98.86(1)	110.65(2)	106.77(1)
PXRD	*P* $\overset{‒}{1}$	1.24	9.2743(5)	10.1495(5)	10.5227(6)	98.72(1)	110.81(1)	106.91(1)

**Table 4:** Comparison of lattice parameters determined at 233 K (single crystal X-ray diffraction data) and 298 K (powder X-ray diffraction data).

2	*D*_x_/g·cm^−3^	Space Group	*a*/Å	*b*/Å	*c*/Å	β/°
SCXRD	1.48	*C*2/*c*	17.4437(3)	7.9305(3)	11.1625(4)	110.131(2)
PXRD	1.47	*C*2/*c*	17.4964(6)	7.9400(3)	11.1614(4)	110.253(3)

**Table 5:** Comparison of lattice parameters determined at 173 K (single crystal X-ray diffraction data) and 298 K (powder X-ray diffraction data).

3	*D*_*x*_/g·cm^−3^	Space Group	*a*/Å	*b*/Å	*c*/Å	β/°
SCXRD	1.28	*P*2_1_/c	7.5109(4)	12.4241(4)	11.6513(4)	106.82(1)
PXRD	1.25	*P*2_1_/c	7.6944(2)	12.5188(5)	11.5183(6)	107.03(1)
